# Vastus medialis motor unit properties in knee osteoarthritis

**DOI:** 10.1186/1471-2474-12-199

**Published:** 2011-09-13

**Authors:** Michael J Berger, David G Chess, Timothy J Doherty

**Affiliations:** 1 School of Kinesiology, Faculty of Health Sciences, The University of Western Ontario, London, ON, Canada; 2 Schulich School of Medicine & Dentistry, The University of Western Ontario, London, ON, Canada; 3 Hand and Upper Limb Centre, St. Joseph's Health Care, The University of Western Ontario, London, ON, Canada; 4 The Departments of Clinical Neurological Sciences and Physical Medicine & Rehabilitation, The University of Western Ontario, London, ON, Canada

## Abstract

**Background:**

Maximal isometric quadriceps strength deficits have been widely reported in studies of knee osteoarthritis (OA), however little is known about the effect of osteoarthritis knee pain on submaximal quadriceps neuromuscular function. The purpose of this study was to measure vastus medialis motor unit (MU) properties in participants with knee OA, during submaximal isometric contractions.

**Methods:**

Vastus medialis motor unit potential (MUP) parameters were assessed in 8 patients with knee OA and 8 healthy, sex and age-matched controls during submaximal isometric contractions (20% of maximum isometric torque). Unpaired t-tests were used to compare groups for demographic and muscle parameters.

**Results:**

Maximum knee extension torque was ~22% lower in the OA group, a difference that was not statistically significantly (p = 0.11). During submaximal contractions, size related parameters of the needle MUPs (e.g. negative peak duration and amplitude-to-area ratio) were greater in the OA group (p < 0.05), with a rightward shift in the frequency distribution of surface MUP negative peak amplitude. MUP firing rates were significantly lower in the OA group (p < 0.05).

**Conclusions:**

Changes in MU recruitment and rate coding strategies in OA may reflect a chronic reinnervation process or a compensatory strategy in the presence of chronic knee pain associated with OA.

## Background

Quadriceps muscle dysfunction in knee osteoarthritis (OA) is well documented, but has been studied predominantly under conditions of maximal activation (i.e. during maximal voluntary isometric contractions) [1]. The activities of daily living are usually performed at submaximal contractile intensities, and further investigation into submaximal neuromuscular function in knee OA is required. Furthermore, the outcomes investigated in knee OA studies are usually restricted to the muscle (e.g. strength, muscle cross-sectional area etc.) and there are few studies measuring neuromuscular parameters.

Quadriceps force required during submaximal and maximal activity is achieved through a combination of motor unit (MU) recruitment and rate coding strategies. In large muscles such as the quadriceps, force increments are achieved predominantly through recruitment of progressively larger MUs, with a smaller contribution from increases in firing rate at higher intensities [2]. MU recruitment and rate coding strategies are altered in models of aging, pain and disease [3,4]. For example, in healthy aging, collateral reinnervation leads to recruitment of larger MUs at reduced firing rates to maintain the same relative contractile intensity compared to younger subjects [5]. Also, experimentally induced joint pain leads to recruitment of different MUs and reduced firing rates compared to a non-painful muscle contraction [6]. Therefore, it could also be affected by the chronic knee pain associated with knee OA. In the only study of MU behaviour in knee OA, Ling et al. reported increases in surface-detected motor unit potential size (S-MUP, reflecting increased MU size) for a given contractile intensity level in those with severe radiographic OA versus controls, with a paradoxical reduction in surface electromyography (EMG) activity and without concomitant changes in firing rate in the vastus medialis [7]. These results require confirmation as radiographic disease severity is only weakly associated with quadriceps muscle strength [8,9] and the severity of symptoms in the severe group was not specified. Furthermore there were proportionally more females in the OA group compared to the control group. Last, reduced surface EMG activity is usually indicative of reduced (not greater) MU recruitment (i.e. a reduction in surface EMG activity is usually interpreted as a reduction in MU recruitment for a given contractile intensity). MU firing rates are also known to be affected by pain [6] and the effect of symptomatic knee OA at the level of the MU has yet to be thoroughly investigated. Therefore, the current study constitutes a preliminary investigation into MU recruitment and firing rate strategies during submaximal voluntary contractions in patients with symptomatic knee OA. We hypothesize that MU recruitment and rate coding strategies will be altered in the presence of chronic knee pain associated with OA.

## Methods

### Study participants

Participants were recruited from a local orthopedic outpatient clinic and included if they met the clinical criteria for knee OA outlined by the American College of Rheumatology [10], had persistent knee pain that required referral to an orthopedic surgeon and received a diagnosis of knee OA from the surgeon based on symptoms and radiographs [11]. Clinical disease severity was measured with the Western Ontario and McMaster Osteoarthritis Index (WOMAC). Exclusion criteria included evidence of any other musculoskeletal, impairment of the lower limbs or previous high tibial ostoetomy or arthroplasty. Ethical approval for the study was obtained from the local institutional ethics review board and written consent was obtained from each participant prior to study commencement. Control subjects were recruited from the local university community and had no self-reported history of knee pain.

### Measurement of isometric muscle torque

Participants were seated upright in a multi-joint dynamometer (Biodex System 3, Shirley, NY), with knee and hip angles of 90° and 100° respectively. The force transducer was positioned with its bottom edge two fingerbreadths proximal to the medial malleolus of the test leg and fixed with a Velcro strap. A seat-belt strap was positioned across the lap in order to avoid unwanted movement. Participants then performed repeated, brief (~5 s) isometric maximal voluntary contractions (MVC) of the quadriceps, each separated by a minimum of 90 s of rest. Maximal contraction intensity was attained when two consecutive MVCs differed by less than 5% (minimum 3 contractions). Visual feedback in the form of the real-time torque tracing and verbal encouragement were provided as motivation. Torque was sampled at 100 Hz, AD converted with a 12-bit converter (CED micro1401 mk II, Cambridge Electronic Design Limited, Cambridge, UK) and displayed in real-time on an online digital system using commercially available software (Spike2 ver. 5, Cambridge Electronic Design).

### Motor unit properties

Decomposition-enhanced quantitative EMG (DQEMG) was used to extract information about individual motor unit potentials (MUPs; which reflect information about the MU) from both an intramuscular concentric needle electrode and surface recording electrodes. The premise of DQEMG is that the complex interference pattern from the intramuscular EMG signal generated during a sustained, submaximal isometric contraction can be decomposed via a series of pattern recognition algorithms, into its constituent individual MUP trains. From these MUP trains, a prototype needle-detected MUP (N-MUP) is extracted that provides quantitative information about MU architecture. The MUP train further serves as a time-locked trigger to extract individual surface motor unit potential (S-MUPs) from the surface signal through spike-triggered averaging. The purpose of extracting S-MUPs from the surface signal is the relatively large recording area of the surface electrodes, compared to the focal needle electrode, provides more valid information about MU size [12]. The DQEMG method has been described in detail previously [13]. Briefly, a series of algorithms decomposes the concentric needle detected EMG signal into its constituent N-MUP trains using information related to both MUP shape and firing times. The firing times of the detected and classified N-MUPs from each train are then used as triggers for locating time-locked 100 ms epochs in the surface signal. These surface signal epochs are then averaged to extract the S-MUP template for a particular MUP train. The N-MUP template is based on a median filtered average of 51 isolated MUPs.

Needle and surface EMG signals were acquired from the vastus medialis muscle using custom DQEMG software on the Neuroscan Comperio system (Neuroscan Medical Systems, El Paso, TX). The vastus medialis was selected because DQEMG has been used previously in this muscle [13], distal vastus medialis muscle fibre morphology has been shown to be altered in end-stage knee OA [14] and needle EMG performed in this muscle is usually less painful than other bellies of the quadriceps. N-MUPs were detected with a commercially available disposable concentric needle electrode (model no. N53153; Teca Corp., Hawthorne, NY). In order to detect S-MUPs, self-adhesive silver/silver chloride electrodes were cut into 1 cm × 3 cm strips and applied over the area of interest, after abrasion of the skin with isopropyl alcohol pads. The active electrode was applied over the motor point of the vastus medialis (3 fingerbreadths superiomedial to the base of the patella). The reference and ground electrodes were applied to the patella and the lateral thigh, respectively. The needle and surface signals were amplified and filtered with a bandpass of 10 Hz to 10 kHz and 5 Hz to 5 kHz, respectively.

Participants were positioned in the dynamometer and the needle was inserted into the muscle belly ~1 cm distal to the active surface electrode at a depth of ~ 0.5-1 cm and held in place manually. Participants were asked to perform a minimal contraction while the needle position was adjusted to minimize the rise times of the N-MUPs from the first 2-3 recruited MUs. With the needle held manually in this position, participants performed repeated contractions at 20% MVC. This intensity was selected because it has been shown to approximate the total population of MUs available [15], participants had difficulty maintaining higher intensity levels for the required 30 s and because the complex interference pattern generated at higher intensities is difficult to accurately decompose and may under represent smaller MUs [16]. A target line was placed across the real-time torque tracing and auditory feedback of the firing pattern was used to ensure maintenance of a steady contraction. Each contraction lasted 30 s, which was the time necessary to allow for an adequate number of averages to adequately extract the S-MUPs. Three to nine contractions were required in order to acquire a sample of at least 20 distinct N-MUPs and S-MUPs for analysis. Needle position and depth were altered to increase the possibility of detecting different N-MUP trains.

**Figure 1:**
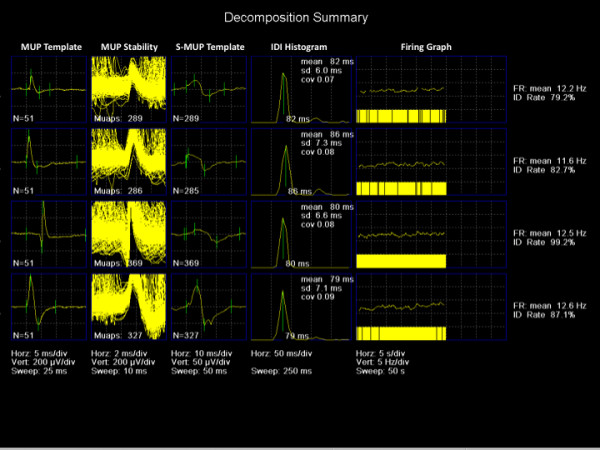
**Output screen for decomposition of the needle EMG interference pattern during a 30 s contraction of the vastus medialis in a healthy control subject**. Five individual motor unit potential trains were identified (labeled 1-5 on the far left). Panel descriptions from left-to-right: MUP Template: individual needle motor unit potential trains representing the median of 51 N-MUPs assigned to the template; MUP Stability: Shimmer plot of the 51 N-MUPs; S-MUP Template: S-MUP derived from spike-triggered averaging of the needle signal; IDI Histogram: Distribution of the IDIs for the N-MUPs comprising the motor unit potential train; Firing Graph: plot of the firing rate characteristics (inverse of the IDI) over the 30 s interval. EMG: electromyography; N-MUP: needle motor unit potential; S-MUP: surface motor unit potential; IDI: interdischarge interval.

Offline analysis of each N-MUP train and S-MUP was performed to determine acceptability. Rejection criteria for an N-MUP train (and its associated S-MUP) included an N-MUP template with less than 51 individual contributions, a non-Gaussian MU interdischarge interval (IDI) histogram, a coefficient of variation greater than 0.3 for the IDI and a non-physiological or inconsistent firing rate [17]. Subsequently, all N-MUP trains and their respective N-MUPs and S-MUPS were visualized and markers for onset, negative-peak, positive-peak and endpoint were readjusted manually when necessary. The firing pattern of each MUP was characterized by a histogram and estimation of the mean interdischarge interval (IDI). Each MU's average firing rate was calculated as the reciprocal of its mean IDI. The DQEMG output for a single contraction from a representative subject is depicted in Figure 1.

### Statistics

Unpaired t-tests were used to compare OA and control groups for demographic and neuromuscular parameters (Graphpad Prism Version 5.0 b, La Jolla, CA). Level of significance was set at p < 0.05.

## Results

Demographic data are presented in Table 1. No significant differences were observed for age or height (p > 0.05), although the larger body mass (p = 0.07) and BMI (p = 0.05) observed in the OA group did approach significance. Disease severity measured by WOMAC score was significantly lower in the control versus OA group (p < 0.05). No significant difference was observed for normalized knee extensor torque (p = 0.11); however, the OA group displayed a non-significant 22% reduction in torque compared to the control group.

**Table 1:** Demographic and needle and surface motor unit potential parameters for OA and control participants

	*Control*	*OA*
*Demographic Parameters*		
Male/Female	4/4	4/4
Age	61.8 ± 5.9	61.3 ± 3.8
Height (m)	1.69 ± 0.07	1.72 ± 0.09
Weight (kg)	77.9 ± 24.0	99.9 ± 20.3
BMI (kg/m^2^)	27.0 ± 27.3	33.4 ± 4.6
WOMAC Score	0.75 ± 0.76	42.4 ± 14.8*
Knee Extensor MVC (N•m/kg)	1.87 ± 0.49	1.47 ± 0.43
*N-MUP Parameters*		
Peak-to-peak voltage (μV)	548 ± 295	505 ± 318
Duration (ms)	10.5 ± 5.1	12.0 ± 4.9*
AAR	1.7 ± 0.5	1.9 ± 0.5*
Turns	2.9 ± 1.2	3.0 ± 1.1
Phases	2.6 ± 0.7	2.6 ± 0.7
*S-MUP Parameters*		
Negative peak amplitude (μV)	41.2 ± 29.2	41.7 ± 29.2
Negative peak duration (ms)	8.9 ± 2.6	9.0 ± 2.4
Negative peak area (μV•ms)	180.4 ± 122.4	173.2 ± 98.7
Firing rate (Hz)	9.1 ± 1.9	8.4 ± 1.8*

Data are presented as means ± standard deviations. BMI: body mass index; WOMAC: Western Ontario and McMaster Osteoarthritis Questionnaire; N-MUP: needle motor unit potential; AAR: area-to-amplitude ratio, S-MUP: surface motor unit action potential.

*Significant difference between Control and OA groups (p < 0.05).

**Figure 2:**
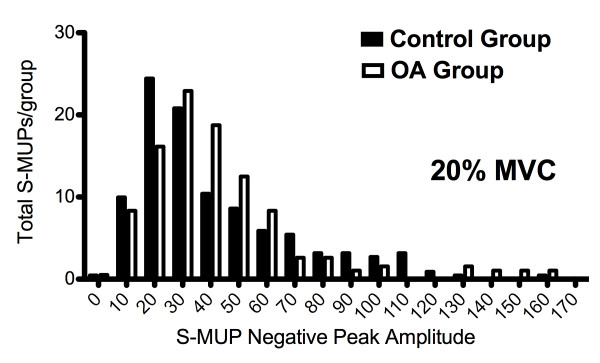
**Distribution of S-MUP amplitudes expressed as total number per bin at 20% MVC for control (open bars) and OA (solid bars) participants**. It appears that the OA participants had a greater number of relatively large S-MUPs, signifying a tendency towards increased MU size. S-MUP: surface motor unit action potential; MU: motor unit.

Size-related parameters of the N-MUPs and S-MUPs, as well as mean MU firing rates were compared (Table 1). The final analysis was conducted on 28 ± 5 (control) and 25 ± 6 (OA) N-MUPs per participant. No significant differences between groups in the number of N-MUPs per participant were noted, thus no single subject was more representative of group statistics than any other (p > 0.05). Mean N-MUP peak-to-peak voltage was not significantly different between groups at either contraction intensity (p > 0.05). A significant increase in N-MUP duration was observed in the OA group. The area-to-amplitude ratio (AAR), also known as N-MUP "thickness" was significantly greater in OA versus control subjects (p < 0.05). N-MUP complexity as measured by the number of turns and phases was not significantly different between groups (p > 0.05). No differences were observed in mean S-MUP size parameters (negative peak amplitude, duration and area) between groups (p > 0.05). However, the frequency distribution of S-MUP amplitudes displayed a rightward shift, with increased numbers of larger amplitudes in the OA group (Figure 2). Firing rates were slightly, but significantly lower in the OA group (p < 0.05).

## Discussion

DQEMG is a fast, valid and reliable method of deriving quantitative information from needle and surface EMG signals [18,19]. N-MUPs and S-MUPs provide information about MU morphology and physiology that is sensitive to pathological processes affecting the size and architecture of the MU [12]. Analysis of the size-related parameters of the MUPs in the OA group in this study reflects recruitment of larger MUs for a given contractile intensity in conjunction with reduced MU firing rates, compared to controls (Table 1). It is unclear whether this change in recruitment strategy reflects a chronic MU remodeling process (i.e. larger MUs due to a collateral reinnervation process [12]), similar to healthy aging [3], or recruitment of larger MUs to maintain a given submaximal contractile intensity in the presence of reduced MU firing rates.

In this study, there was no difference in N-MUP peak-to-peak voltage between OA and control groups (Table 1). N-MUP amplitude represents the contribution of only those active muscle fibres detected within close proximity of the recording electrode and is affected by factors such as temporal dispersion and variation in terminal axon conduction velocity [12]. Other N-MUP parameters such as duration and AAR, or "thickness" are not as affected by electrode position and provide more robust information about MU size [12]. Increases in duration and thickness suggest that larger numbers of muscle fibres are contributing to the resultant MUP. We observed that thickness and duration were significantly larger in the OA group (Table 1). Alternatively, mean S-MUP amplitudes, which are thought to be the parameter the best represents MU size, [17,20] were not significantly different between groups (Table 1). This lack of between-group difference in S-MUP negative peak amplitude could indicate similarly sized MUs, however there was large variability in S-MUP negative peak amplitude, which may have diluted any true difference. Electrophysiological studies from studies of healthy older adults suggest that MU remodeling is a chronic process, often requiring decades to manifest [1]. In this regard, it is possible that MU remodeling is occurring in patients with OA, but the extent of the remodeling is not substantive in this cohort to result in mean S-MUPs size differences. In support of this hypothesis, we present the frequency distribution of S-MUP amplitudes for OA and control participants in Figure 2, which illustrates a rightward shift in the S-MUP size distribution with increased numbers of larger S-MUPs in the patients. In a previous study we speculated that a qualitative rightward shift in the frequency distribution of S-MUP amplitudes indicates that only select MUs had increased in size through collateral reinnervation [17].

Alternatively, recruitment of larger MUs may be a compensatory process to account for the reduced MU firing rates observed in the OA group (Table 1). Increased number of muscle fibres per MU results in increased torque for that MU [21]. In this scenario, a larger MU would be required to maintain a given contractile intensity in the setting of reduced firing rates (i.e. a matching of rate coding to recruitment to maintain a given contractile intensity). Reduced MU firing rates could be explained by the significant joint pain experienced by the OA group compared to the controls. It has been shown in experimental pain models that MU firing rate and MU recruitment strategy are altered [6]. Tucker et al. injected the infrapatellar fat pad with hypertonic saline to induce pain and reported reduced MU firing rates and a greater contribution from more MUs to maintain submaximal force [6]. The significance of such small reductions in MU firing rate between OA and control participants (< 1 Hz) is questionable (Table 1), however larger muscles such as the vastus medialis have been shown to grade force through recruitment as opposed to rate coding strategies, such that step increases in contractile intensity are accomplished predominantly through increases in recruitment [21]. Accordingly, MUs in this muscle have been shown to have a narrow range of firing rates (approximately 8-26 Hz) [21]. In such a narrow window, it's possible that even small changes in firing rates may indicate significant changes in the motor control strategy employed by this muscle. Furthermore, the small absolute reductions in firing rate observed in this study are consistent with studies reporting reduced firing rates in experimentally induced pain [6,22].

It is surprising that no difference in normalized strength between healthy controls and OA subjects was observed, as muscle weakness has been uniformly reported in knee OA studies (Table 1) [1]. As the goal of this experiment was obtain information about MU properties, only a small number of participants were recruited due to the ability to sample large numbers of MUs within each participant. It is likely that low sample size led to type II error in this portion of the study as there was a non-significant ~22% difference in maximal knee extensor torque between groups. This falls within the range of strength deficits reported previously for studies of knee extensor torque in knee OA [1].

A limitation of this study is that we did not obtain information about MUs recruited at higher contraction intensities. Pilot testing revealed that many participants could not maintain a contraction intensity ≥ 30% MVC for 30 s. Unfortunately, it is difficult both technically as well as physically (i.e. from the perspective of the patient) to contract and sample MUs at higher contractile intensities in this muscle. McNeil et al. reported that a contractile intensity of ~25% MVC best reflected the diversity of the MU pool in the tibialis anterior, while contractions at relatively low (< 10% MVC) and high intensities (> 40% MVC) yielded undersampling of larger and smaller MUs respectively [15]. Furthermore, a more complex interference pattern generated at higher contractile intensities (due to a greater number of larger active MUs) results in a greater number of MUP superimpositions, reducing the probability of accurately sampling smaller MUs [16]. Thus, we contend that the 20% MVC intensity used in this study provides a reasonable sampling of the entire MU pool. Another limitation of this study is the large variability in clinical severity as measured by WOMAC score, in the OA group (coefficient of variation = ~35%, Table 1). It is possible that there is an association between symptom severity and the magnitude of MU changes, however we did not possess the necessary study power to undertake this analysis. Future studies should examine the relationship between symptom severity and quadriceps neuromuscular changes in this population.

## Conclusion

These preliminary results suggest that MU recruitment and firing rate are altered in patients with knee OA. It remains to be determined whether these changes are due to permanent MU remodeling or reflect a compensatory recruitment strategy in response to OA symptoms or structural changes. The clinical significance of these findings is unclear, but pathological changes to the MU may impact on function and disease prognosis.

## Competing interests

The authors declare that they have no competing interests.

## Authors' contributions

MJB was responsible for study design, subject recruitment, data collection and analysis and drafting of the final manuscript. DGC contributed to study design and subject recruitment. TJD was responsible for study design, data analysis and interpretation of the data. All authors gave final approval for submission of the manuscript.

## Pre-publication history

The pre-publication history for this paper can be accessed here:

http://www.biomedcentral.com/1471-2474/12/199/prepub

## References

[B1] Berger MJ Doherty TJ Sarcopenia: Prevalence, Mechanisms, and Functional Consequences Interdiscip Top Gerontol 2010 37 94 114 20703058 10.1159/000319997

[B2] Kukulka CG Clamann HP Comparison of the recruitment and discharge properties of motor units in human brachial biceps and adductor pollicis during isometric contractions Brain Res 1981 219 45 55 10.1016/0006-8993(81)90266-3 7260629

[B3] Gordon T Hegedus J Tam SL Adaptive and maladaptive motor axonal sprouting in aging and motoneuron disease Neurol Res 2004 26 174 185 10.1179/016164104225013806 15072637

[B4] Duchateau J Semmler JG Enoka RM Training adaptations in the behavior of human motor units J Appl Physiol 2006 101 1766 1775 16794023 10.1152/japplphysiol.00543.2006

[B5] Roos MR Rice CL Vandervoort AA Age-related changes in motor unit function Muscle Nerve 1997 20 679 690 10.1002/(SICI)1097-4598(199706)20:6<679::AID-MUS4>3.0.CO;2-5 9149074

[B6] Tucker KJ Hodges PW Motoneurone recruitment is altered with pain induced in non-muscular tissue Pain 2009 141 151 155 10.1016/j.pain.2008.10.029 19095357

[B7] Ling SM Conwit RA Talbot L Shermack M Wood JE Dredge EM Weeks MJ Abernethy DR Metter EJ Electromyographic patterns suggest changes in motor unit physiology associated with early osteoarthritis of the knee Osteoarthritis Cartilage 2007 15 10 1134 40 10.1016/j.joca.2007.03.024 17543548 PMC2259251

[B8] O'Reilly SC Jones A Muir KR Doherty M Quadriceps weakness in knee osteoarthritis: the effect on pain and disability Ann Rheum Dis 1998 57 588 594 10.1136/ard.57.10.588 9893569 PMC1752483

[B9] Baker KR Xu L Zhang Y Nevitt M Niu J Aliabadi P Yu W Felson D Quadriceps weakness and its relationship to tibiofemoral and patellofemoral knee osteoarthritis in Chinese: the Beijing osteoarthritis study Arthritis Rheum 2004 50 1815 1821 10.1002/art.20261 15188358

[B10] Altman R Asch E Bloch D Bole G Borenstein D Brandt K Christy W Cooke TD Greenwald R Hochberg M Development of criteria for the classification and reporting of osteoarthritis. Classification of osteoarthritis of the knee. Diagnostic and Therapeutic Criteria Committee of the American Rheumatism Association Arthritis Rheum 1986 29 1039 1049 10.1002/art.1780290816 3741515

[B11] Gossec L Paternotte S Maillefert JF Combescure C Conaghan PG Davis AM Gunther KP Hawker G Hochberg M Katz JN The role of pain and functional impairment in the decision to recommend total joint replacement in hip and knee osteoarthritis: an international cross-sectional study of 1909 patients. Report of the OARSI-OMERACT Task Force on total joint replacement Osteoarthritis Cartilage 2011 19 147 154 10.1016/j.joca.2010.10.025 21044689 PMC4151518

[B12] Stalberg E Nandedkar SD Sanders DB Falck B Quantitative motor unit potential analysis J Clin Neurophysiol 1996 13 401 422 10.1097/00004691-199609000-00004 8897206

[B13] Doherty TJ Stashuk DW Decomposition-based quantitative electromyography: methods and initial normative data in five muscles Muscle Nerve 2003 28 204 211 10.1002/mus.10427 12872325

[B14] Fink B Egl M Singer J Fuerst M Bubenheim M Neuen-Jacob E Morphologic changes in the vastus medialis muscle in patients with osteoarthritis of the knee Arthritis Rheum 2007 56 3626 3633 10.1002/art.22960 17968889

[B15] McNeil CJ Doherty TJ Stashuk DW Rice CL The effect of contraction intensity on motor unit number estimates of the tibialis anterior Clin Neurophysiol 2005 116 1342 1347 10.1016/j.clinph.2005.02.006 15876553

[B16] Boe SG Stashuk DW Brown WF Doherty TJ Decomposition-based quantitative electromyography: effect of force on motor unit potentials and motor unit number estimates Muscle Nerve 2005 31 365 373 10.1002/mus.20266 15627267

[B17] Boe SG Stashuk DW Doherty TJ Motor unit number estimates and quantitative motor unit analysis in healthy subjects and patients with amyotrophic lateral sclerosis Muscle Nerve 2007 36 62 70 10.1002/mus.20784 17455264

[B18] Boe SG Antonowicz NM Leung VW Shea SM Zimmerman TC Doherty TJ High inter-rater reliability in analyzing results of decomposition-based quantitative electromyography in subjects with or without neuromuscular disorder J Neurosci Methods 2010 192 1 138 45 10.1016/j.jneumeth.2010.07.024 20674605

[B19] Boe SG Stashuk DW Doherty TJ Motor unit number estimation by decomposition-enhanced spike-triggered averaging: control data, test-retest reliability, and contractile level effects Muscle Nerve 2004 29 693 699 10.1002/mus.20031 15116373

[B20] Stalberg E Fawcett PR Macro EMG in healthy subjects of different ages J Neurol Neurosurg Psychiatry 1982 45 870 878 10.1136/jnnp.45.10.870 7143007 PMC491590

[B21] Roos MR Rice CL Connelly DM Vandervoort AA Quadriceps muscle strength, contractile properties, and motor unit firing rates in young and old men Muscle Nerve 1999 22 1094 1103 10.1002/(SICI)1097-4598(199908)22:8<1094::AID-MUS14>3.0.CO;2-G 10417793

[B22] Tucker K Butler J Graven-Nielsen T Riek S Hodges P Motor unit recruitment strategies are altered during deep-tissue pain J Neurosci 2009 29 10820 10826 10.1523/JNEUROSCI.5211-08.2009 19726639 PMC6665526

